# The impact of Asic2 deletion on metabolic homeostasis in mice

**DOI:** 10.14814/phy2.70514

**Published:** 2025-09-12

**Authors:** Madison Hamby, Jasleen Kaur, Seth Lirette, Jussara M. do Carmo, Joshua Speed, Tawhida Islam, Emily Hildebrandt, Elizabeth Barr, Lavanya Challagundala, Heather A. Drummond

**Affiliations:** ^1^ Department of Physiology and Biophysics University of Mississippi Medical Center Jackson Mississippi USA; ^2^ Department of Cell and Molecular Biology and Genomics Core Facility University of Mississippi Medical Center Jackson Mississippi USA; ^3^ Department of Data Science University of Mississippi Medical Center Jackson Mississippi USA

**Keywords:** degenerin, diet induced obesity, energy balance

## Abstract

Acid‐Sensing Ion Channel 2 (Asic2) is expressed in many brain regions, including the hypothalamus, the homeostatic center for hunger‐satiety‐energy expenditure. We used the HypoMap reference atlas of the mouse hypothalamus to localize Asic2 message in the specific hypothalamic regions and found that Asic2 is the predominant degenerin subunit expressed in mouse hypothalamic neurons in the ventromedial, lateral, and arcuate regions and is expressed in pro‐opiomelanocortin (Pomc), agouti‐related peptide (Agrp), as well as leptin (Lepr) and insulin receptors (Insr) positive neurons. Thus, we investigated if Asic2 plays an important role in body weight control and energy expenditure before and after high‐fat diet (HFD). Asic2^−/−^ male mice had lower lean body mass. On normal chow, Asic2^−/−^ mice consumed more food, displayed elevated total energy expenditure, and a higher respiratory exchange ratio compared to WT mice. On the HFD, Asic2^−/−^ female mice had greater motor activity and resting and total energy expenditure. We found no effect of genotype on plasma glucose, insulin, or ghrelin levels; however, leptin was elevated in male Asic2^−/−^ mice following the HFD. Our findings suggest that Asic2 plays an important role in metabolic homeostasis, possibly through regulation of central pathways involved in energy balance.

## 1 INTRODUCTION

Obesity is a risk factor for cardiovascular disease, and the incidence of obesity and related metabolic disease in the United States is on the rise (Chen & Stinnett, 2008). Obesity is a disruption of metabolic homeostasis resulting from an imbalance of satiety‐hunger signals, energy intake, and expenditure that leads to the development of cardiometabolic syndrome (da Silva et al., 2021; do Carmo et al., 2019; Garfield et al., 2015; Lavoie et al., 2023). Degenerin ion channels are a family of cation channels that include Epithelial Na^+^ Channel (ENaC) and Acid Sensing Ion Channel (Asic) groups and are expressed in many of the cell types involved in the pathology of metabolic syndrome, including epithelial, endothelial, vascular smooth muscle, immune cells, and neurons (Barbaro et al., 2017; Drummond, 2021; Drummond et al., 2001; Kellenberger & Schild, 2002; Pearce, 2003; Pochynyuk et al., 2007; Syntichaki & Tavernarakis, 2004). However, the role of degenerin channels in the development of metabolic disease is relatively unknown. We recently showed that mice lacking normal levels of Asic2 and βENaC were unexpectedly protected from weight gain, metabolic disruption, and hepatic steatosis when placed on a 10‐week 60% kcal high‐fat diet (HFD) (Hamby et al., 2024). These unanticipated findings raised the possibility that degenerin proteins may contribute to metabolic homeostasis.

Many degenerin channels operate as sensors of the extracellular environment. ENaC members of this family can act as Na^+^ sensors in the renal cortical collecting ducts, shear stress sensors in endothelial cells, stretch/displacement sensors in vascular smooth muscle cells, arterial baroreceptors, and peripheral touch neurons. Asic members may act as extracellular H^+^ sensors in peripheral and central neurons and peripheral arterial chemoreceptors (Chung et al., 2011, 2013; Drummond et al., 2001; Fronius & Clauss, 2008; Lu et al., 2009; Page et al., 2004; Price et al., 2000; Shi et al., 2013; Tan et al., 2007). As sensors, degenerins are important in homeostatic regulation; they contribute to local control of blood flow (myogenic autoregulation), arterial baroreflex control of blood pressure, renal salt and water balance, and circadian rhythm of temperature (Gannon et al., 2015; Grifoni et al., 2010; Lu et al., 2009; McDonald et al., 1999; Peng et al., 2023; Pradervand et al., 1999). Whether degenerin channels participate in sensing the metabolic environment and regulating metabolic homeostasis is unknown.

In mammals, the hypothalamus is a metabolic homeostatic center and integrates hormonal signals of hunger and satiety (Belgardt et al., 2009; Lavoie et al., 2023; Tu et al., 2022). While multiple regions are involved in sensory input, central integration, and execution of the behavioral response to hunger and satiety, the pro‐opiomelanocortin (Pomc) and agouti‐related peptide (Agrp)/neuropeptide Y (Npy) containing neurons of the arcuate region play a critical role in metabolic homeostasis. Pomc neurons are important in signaling satiety since Pomc deletion causes weight gain by increasing food consumption and lowering oxygen consumption, thereby increasing obesity susceptibility (Challis et al., 2004). Agrp neurons are important in signaling hunger. Agrp deletion in adult mice leads to starvation, and conditional deletion in gabaergic neurons lowers body mass and increases oxygen consumption, motor activity, and protects against HFD‐induced obesity (Tong et al., 2008). Npy can co‐express with Agrp, and its deletion also suppresses feeding and body weight (Herzog, 2003). However, whether Asic or related Scnn1 channels are expressed in arcuate neurons was unknown until the publication of the HypoMap data set by Steuernagel et al. (2022).

The HypoMap data set is a searchable, publicly available, reference map of the mouse hypothalamus (Steuernagel et al., 2022). The map was generated by integrating 18 single cell RNA sequencing studies into a single data base. Using the online user‐friendly explorer tool available at https://cellxgene.cziscience.com/collections, we identified expression of Asic2 and other degenerins throughout multiple hypothalamic regions important in homeostasis. These initial findings led us to further examine specific aspects of degenerin expression in the hypothalamus using Seurat in R Studio. In the current study, we provide evidence of degenerin expression in hypothalamic cells and regions using the HypoMap data set. We also provide co‐expression evidence of Asic2, the predominant degenerin expressed in the arcuate as well as the entire hypothalamus, in neurons expressing Agrp, Npy, and Pomc. Finally, we examined food consumption responses to leptin and metabolic homeostasis in global Asic2 knockout mice. Our findings demonstrate an important role for Asic2 in metabolic homeostasis and the need for additional cell‐specific knockout mice to investigate the neuron population‐specific role of Asic2 in hunger‐satiety‐energy homeostasis.

## 2 METHODS

### 2.1 Analysis of degenerin expression in HypoMap data set

**FIGURE 1:**
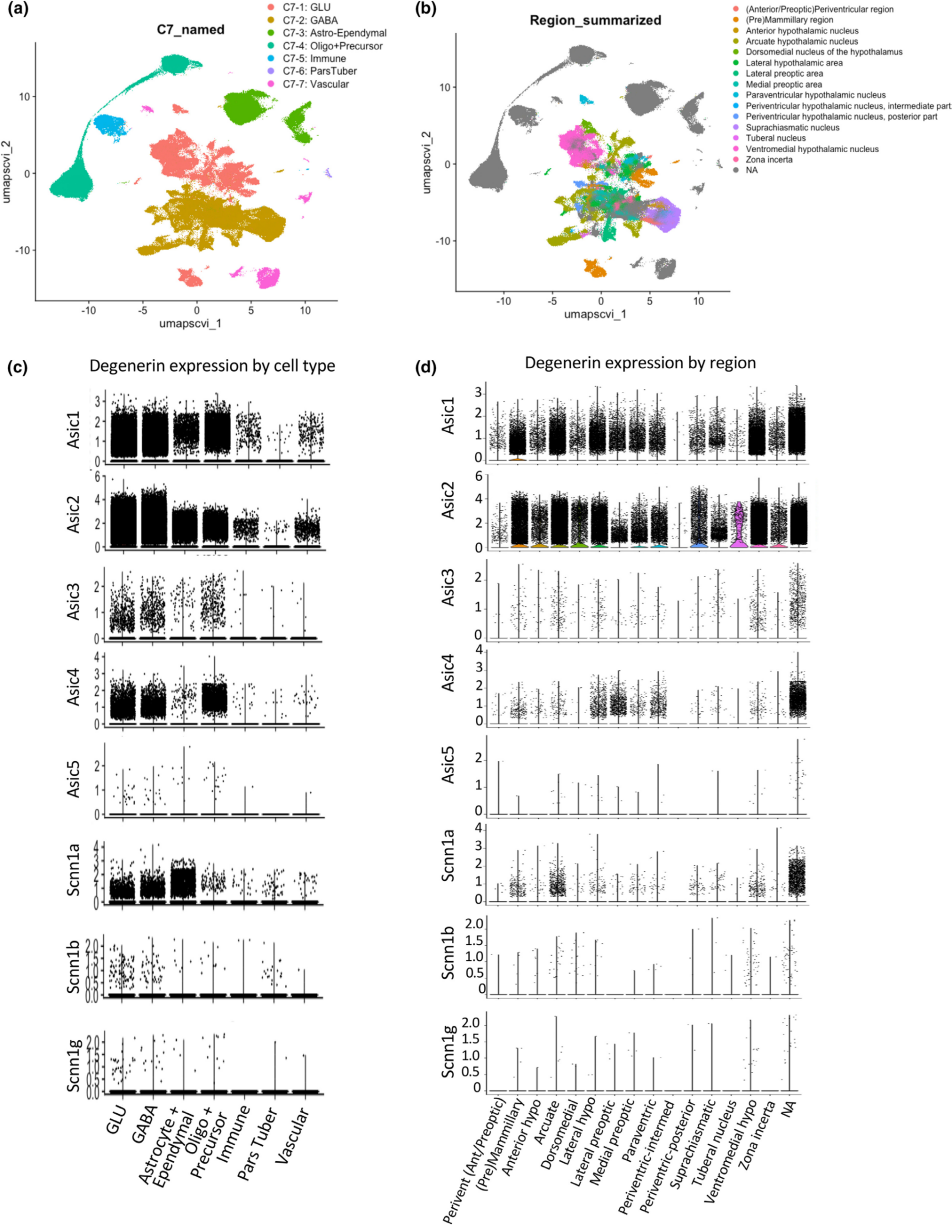
Seurat analysis of HypoMap dataset to determine degenerin subunit expression in the mouse hypothalamus. The HypoMap data set and a user‐friendly explorer tool are available at https://cellxgene.cziscience.com/collections. RDS file was downloaded from the Apollo Repository hosted by Cambridge University (https://www.repository.cam.ac.uk/items/8f9c3683‐29fd‐44f3‐aad5‐7acf5e963a75). (a) Dimensional reduction plot of cluster identification by cell type (“C7_named”). (b) Dimensional reduction plot identifying region (“Region_summarized”). (c) Violin plot showing expression of mammalian degenerins in different cell types. These data show degenerins are expressed in all cell types but are expressed to the greatest extent in glutamatergic and gabaergic neurons, followed by astrocytes and oligodendrocytes. (d) Violin plots showing expression of mammalian degenerins in 16 regions. “NA” refers to cells where no marker was present to identify localization, that is, oligodendrocytes, astrocytes, tanycytes, and ependymal cells. UMAP, universal manifold approximation and projection. Script: (1A) DimPlot(hypoMap, group.by = “C7_named”, label = FALSE). (1B) DimPlot(hypoMap, group.by = “Region_summarized”, label = FALSE). (1C) VlnPlot(object = hypoMap, features = “Asic1”, group.by = “C7_named”). (1D) VlnPlot(object = hypoMap, features = “Asic1”, group.by = “Region_summarized”). #Other gene target names: Asic2, Asic3, Asic4, Asic5, Scnn1a, Scnn1b, Scnn1g.

**FIGURE 2:**
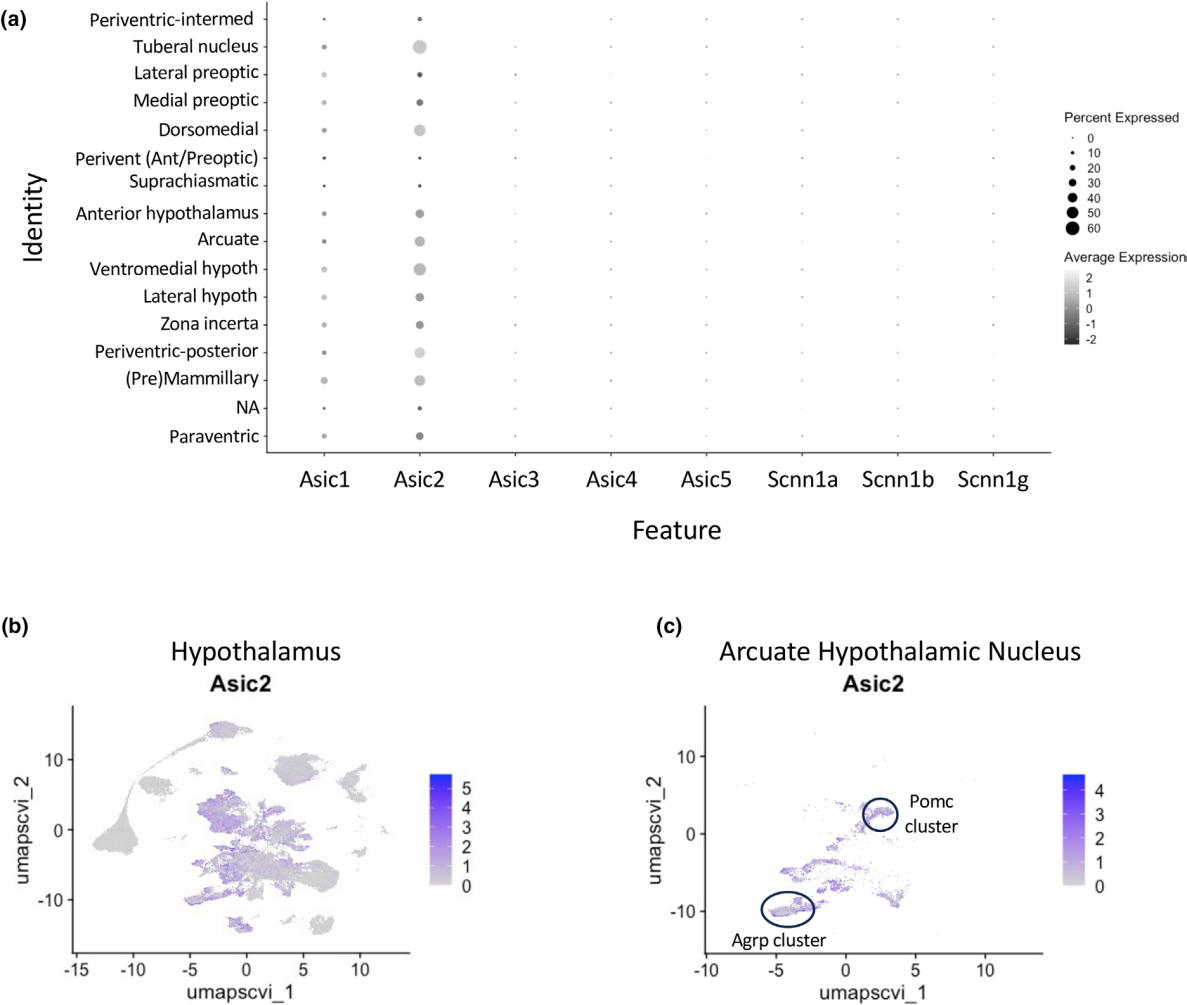
(a) A dot plot showing the percent neurons expressing Asic1‐5 and Scnn1a, b, and g in hypothalamic regions. Asic2, followed by Asic1, is the most abundantly expressed degenerin message in the mouse hypothalamus, with both Asic1 and Asic2 predominantly expressed in the premammillary, arcuate, lateral hypothalamus, and ventromedial hypothalamus regions. The periventricular‐intermediate region has the fewest Asic1/2 positive cells. “NA” refers to cells where no marker was present to identify localization, that is, oligodendrocytes, astrocytes, tanycytes, and ependymal cells. (b) Feature plot of Asic2 expression in the hypothalamic cell population. (c) Feature plot of Asic2 expression in the arcuate neuron population. Argp and Pomc clusters are identified. Script: (2A) DotPlot(hypoMap, features = “Asic1”, split.by = “Author_Region”, cols = “Greys”). #Individual DotPlots also generated for Asic2, Asic3, Asic4, Asic5, Scnn1a, Scnn1b, Scnn1g. (2B) FeaturePlot(hypoMap, feature = “Asic2”, raster = TRUE, raster.dpi =c(512, 512), label = FALSE). (2C) hypoMap <‐ SetIdent(hypoMap, value = “Region_summarized”). AHN_subset <‐ subset(x = hypoMap, idents = “Arcuate hypothalamic nucleus”). FeaturePlot(AHN_subset, features = “Asic2”, raster = TRUE, raster.dpi =c(512, 512), label = FALSE).

The HypoMap data set is a unified hypothalamic reference map created by the integration of 18 publicly available single‐cell RNA sequencing data sets and includes 384,925 cells (Steuernagel et al., 2022). The data set and an explorer tool are available at https://cellxgene.cziscience.com/collections. The RDS file was downloaded from https://www.repository.cam.ac.uk/items/8f9c3683‐29fd‐44f3‐aad5‐7acf5e963a75 and is available from the Apollo Repository hosted by the University of Cambridge. The RDS file was analyzed using Seurat 5 in R Studio (Version 2024.04.2 + 764). We generated dimensional reduction, or Uniform Manifold Approximation and Projection (UMAP), clusters that are labeled for cell type based on the “C7_named” clustering level and regional markers (“Region_summarized”) in the meta data (Steuernagel et al., 2022). To identify the cell types and hypothalamic regions expressing the mammalian degenerins, we generated violin plots and a dot plot for Asic1–5 and Scnn1a, b, and g. We generated feature plots of Asic2 in the entire hypothalamus and arcuate region subset. In the dimensional reduction plots, violin plots, and feature plots in Figures 1 and 2, each dot represents a single cell.

### 2.2 Analysis of the metabolic homeostasis of the Asic2 knockout mouse model

#### 2.2.1 Experimental design of animal studies

All animal work was done under the Association for Assessment and Accreditation of Laboratory Animal Care (AAALAC) and the University of Mississippi Medical Center (UMMC) Institutional Animal Care and Use Committee (IACUC). The Asic2 global knockout model has been maintained in our laboratory group for 20 years. The model is available through Jackson Labs (Asic2^−/−^; RRID: IMSR_JAX:013126). Knockout (Asic2^−/−^) and wildtype (Asic2^−/−^/WT) mice, generated from Asic2^+/−^ mating pairs, were maintained as homozygous mating pairs. Mice were maintained under a 12‐h light/dark cycle and permitted food and water ad libitum. Adult male and female mice were used in these studies. Post‐analysis genotypes were confirmed in all animals using liver DNA. All protocols were approved by the Institutional Animal Care and Use Committee at UMMC.

#### 2.2.2 Food consumption response to leptin

All mice were raised on standard normal chow (14% kcal fat, 54% kcal carbohydrate, Envigo, Cat #TD.8604). We examined food consumption in response to intraperitoneal saline (0.9% NaCl, 300 μL) or leptin injection (1 then 2 μg/g body weight on consecutive days, *n* = 4/group) in a group of mice of similar age (14–16 weeks of age, *n* = 4 females/group). Food consumption was measured at 30 min, 2 h, and 4 h following intraperitoneal saline and intraperitoneal leptin. Body mass was measured prior to injection and again the morning following leptin injections.

#### 2.2.3 Metabolic and behavioral phenotyping

To determine the metabolic and behavioral phenotype of Asic2^−/−^ mice, a separate group of 16‐week‐old male and female wildtype and Asic2^−/−^ mice (*n* = 7–8/group) were weighed and then placed in the Sable Systems Promethion CoreTM metabolic phenotyping system for 5 days housed in our animal facility at 23°C. This system provides continuous high‐resolution measurements of oxygen consumption and carbon dioxide production. Mice were housed individually in temperature‐controlled metabolic cages with ad libitum access to food and water and were allowed to acclimate for 48 h, followed by 72 h of recording. Energy expenditure (kcal/day) was calculated from VO_2_ and VCO_2_ data using the Weir equation 3.941 × VO_2_ (L/day) + 1.106 × VCO_2_ (L/day). These values were integrated over time using MetaScreen software provided by Sable Systems. To estimate basal metabolic rate, we used the mean energy expenditure during the lowest energy expenditure 30‐min period (R_EE_30 in Promethion Data Analysis Guide). Total food consumption was the average mass of food removed from the food bin in 24 h. Food consumption, motor activity, sleep, and body mass were recorded. These values were integrated over time using MetaScreen software provided by Sable Systems.

Upon removal from the metabolic cages, body composition was assessed using quantitative magnetic resonance imaging (4‐in‐1 EchoMRI‐900TM, Echo Medical System, Houston, TX). Access to the Echo MRI and Promethion system was supported by the Imaging Core and Metabolic Phenotyping Core, respectively, in the Department of Physiology and Biophysics at UMMC. Mice were returned to their home cages, placed on a HFD (60% kcal from fat, 21% kcal from carbohydrate, Envigo, Cat #TD.06414) for 5 weeks; then metabolic and behavioral phenotyping and body composition measurements were reassessed. Upon completion of the study, mice were fasted for 4 h; blood and organs were collected, weighed, then snap‐frozen in liquid nitrogen and stored at −70°C. Livers were prepared for EchoMRI and Oil Red O staining as described below. To obtain body length, we measured the distance from the tip of the nose to the base of the tail using a mm ruler. To obtain tibial length, we measured the distance from the ankle to the knee using a mm ruler.

#### 2.2.4 Plasma assays

Plasma leptin (R&D Systems, Cat #MOB00B; RRID:AB_2943468), ghrelin (Millipore/Sigma Cat #EZRGRT‐91K), and insulin (Crystal Chem, Cat #90080; RRID:AB_2783626) were assessed by ELISA, and glucose was assessed using a VetAxel Chemical Analyzer. These assays were conducted by the Analytical and Assay Core in the Department of Physiology and Biophysics at UMMC. The Analytical and Assay Core uses a standardized approach and internal standards to provide a repeatable and reliable assessment.

#### 2.2.5 Liver fat accumulation

Whole livers were weighed and assessed for fat content using EchoMRI, then fractioned for fixation in 4% paraformaldehyde for cryosections and Oil Red O staining through the Histology Core in the Department of Physiology and Biophysics at UMMC (Badmus et al., 2023; Hamby et al., 2024; Stec et al., 2019). The remaining liver samples were stored at −70°C. Genotype confirmation and triglyceride content were determined later (AbCam Cat #65336).

### 2.3 Quantification and statistical analysis

All metabolic and behavioral data are presented as the average of 3 24‐h periods in 6–7 animals/group. Data were prepared in Excel. Body composition and liver adiposity data were exported to GraphPad Prism for statistical analysis. Due to differences in body masses among groups, covariate adjusted analyses were conducted in STATA for metabolic‐behavioral, plasma hormone, and morphometric data using generalized linear models with appropriate families (Gaussian for normal data and gamma for right‐skewed data) with fully interacted genotype/sex/diet variables. Marginal means and their associated effects were then estimated from these models. Because all estimates come from a single model and are estimated from a pooled post‐stratification, the type‐I error rate is controlled and no further post hoc adjustments are necessary. Modified means, *p* values, and effect sizes for the covariates with the largest effect size are provided in table format in figure with graphical presentation of absolute values. The absolute data and adjusted means do not align for all measurements. Thus, the covariate analyses were used to discuss outcomes. Other data were analyzed using two‐way repeated measures ANOVA (body composition and food consumption with leptin) or two‐way ANOVA (liver fat content and plasma hormones). Both ANOVAs were followed by a Fisher LSD Post Hoc Analysis where comparisons were identified a priori and limited to between genotype comparisons within each sex. *p* values for differences between genotypes where *p* < 0.200 are provided in figure panels. Data represent mean ± standard error of the mean.

## 3 RESULTS

### 3.1 Seurat analysis of the HypoMap data set

Figure 1a is a dimension reduction plot that shows the UMAP clusters identified by cell type (“C7_named”). Figure 1b identifies the regional markers associated with the neurons (“Region_summarized”) (Steuernagel et al., 2022). Non‐neuronal cells and a small population of neuronal cells that do not express region‐specific markers are colored gray. To summarize expression among the cell types (Figure 1c) and hypothalamic regions (Figure 1d), we generated violin plots for Asic1–5, Scnn1a, b, and g. A dot plot for hypothalamic regions (Figure 2a) more starkly compares the percent of neurons expressing Asic1 and Asic2. Neuronal transcript abundance in the hypothalamus is as follows: Asic2 > Asic1 > Asic4 > Scnn1a > Asic3 > Scnn1b > Scnn1g ~ Asic5, with similar numbers of glutamatergic and gabaergic neurons. Asic1 and Asic2 are also expressed in a large population of astrocytes and ependymal cells, oligodendrocytes, and oligodendrocyte precursor cells. Scnn1a is also expressed in a large population of astrocyte/ependymal cells. The greatest density of Asic1 and Asic2 expressing cells is in the pre‐mammillary, arcuate, lateral hypothalamus, and ventromedial hypothalamus regions. The latter three regions play an important role in metabolic homeostasis regulating hunger‐satiety, feeding behavior, and motor activity. Asic2 is expressed in more cells than Asic1 in these regions (Figure 2). Feature plots visualizing Asic2 expression in the hypothalamus (Figure 2b) and arcuate region (Figure 2c) are shown with the position of the Agrp and Pomc clusters indicated. Table 1 summarizes the co‐expression rate of Asic2 in Agrp, Pomc, Lepr, Insr, Npy, and Asic1 positive neurons in all arcuate neurons. Table 1 further shows the co‐expression rate of Asic2 and Lepr, Insr, Npy, and Asic1 within Agrp or Pomc arcuate neuron populations. Within the arcuate region, Asic2 is expressed in approximately 50% of Agrp and Pomc neurons, 60%–65% of Agrp or Pomc neurons co‐expressing Lepr, and 73%–66% of Agrp or Pomc neurons expressing Insr. In contrast, Asic2 only co‐expresses with Npy in 30% of arcuate neurons. These findings suggest that Asic2 tends to be expressed in a select population of metabolically active arcuate neurons. Asic2 is expressed in 53% of Asic1 arcuate neurons, raising the possibility that Asic1 and Asic2 may form heteromeric channels in some neurons. We also examined the expression rate of the metabolic markers (Pomc, Agrp, Lepr, and Insr) in Asic2 positive arcuate neurons and found much lower co‐expression rates, suggesting that Asic2 is preferentially found in arcuate neurons expressing metabolic markers.

**TABLE 1:** Co‐expression rates of Asic2 with Agrp, Pomc, Lepr, Insr, Npy, and Asic1 in arcuate neurons in the HypoMap dataset. The table also includes co‐expression of the markers within the Asic2 positive arcuate neurons, which show Asic2 is preferentially expressed with the most markers, rather than the markers preferentially expressing in Asic2 positive cells. The right side of the table provides co‐expression rates of Asic2 with Lepr, Insr, Npy, and Asic1 *within* the Agrp and Pomc positive arcuate neuron populations, which suggest Asic2 is more likely to be expressed in Agrp and Pomc neurons that also express Insr or Lepr, respectively. Notably, Asic2 has the highest co‐expression rate in Agrp^+^ plus Lepr^+^ neurons. Of the markers listed, the lowest co‐expression rate was in Npy positive arcuate neurons.

Asic2—metabolic marker co‐expression rates (%) HypoMap						
Neuron metabolic marker (MM)	All arcuate neurons		Agrp^+^ arcuate neurons		Pomc^+^ arcuate neurons	
	Asic2 in MM^+^ neurons	MM in Asic2^+^ neurons	Asic2 in MM^+^ neurons	MM in Asic2^+^ neurons	Asic2 in MM^+^ neurons	MM in Asic2^+^ neurons
Agrp^+^	47	26	47	100		
Pomc^+^	46	31			46	100
Lepr^+^	60	12	60	22	65	19
Insr^+^	67	31	73	34	66	33
Npy^+^	30	20	32	41	36	20
Asic1^+^	53	30	64	16	56	21

*Note*: Script: hypoMap <‐ SetIdent(hypoMap, value = “Region_summarized”). AHN_subset <‐ subset(x = hypoMap, idents = “Arcuate hypothalamic nucleus”). Idents (AHN_subset, WhichCells(object = AHN_subset, expression = Npy > 0, slot = 'data')) <‐ 'Npy_positive'. Idents(AHN_subset, WhichCells(object = AHN_subset, expression = Asic2 > 0, slot = 'data')) <‐ 'Asic2_positive'. #for next line where features=(c, gene1, gene2); will assess expression of gene1 in gene2 positive cells.... percentage=DotPlot(AHN_subset, features= c(“Npy”, “Asic2”), cols=c(“green”, “red”)). percentage$data. #Other target names included Pomc, Agrp, Insr, Lepr, Asic1.

Abbreviation: MM, metabolic marker.

### 3.2 Leptin on food consumption in normal fat‐fed mice

**FIGURE 3:**
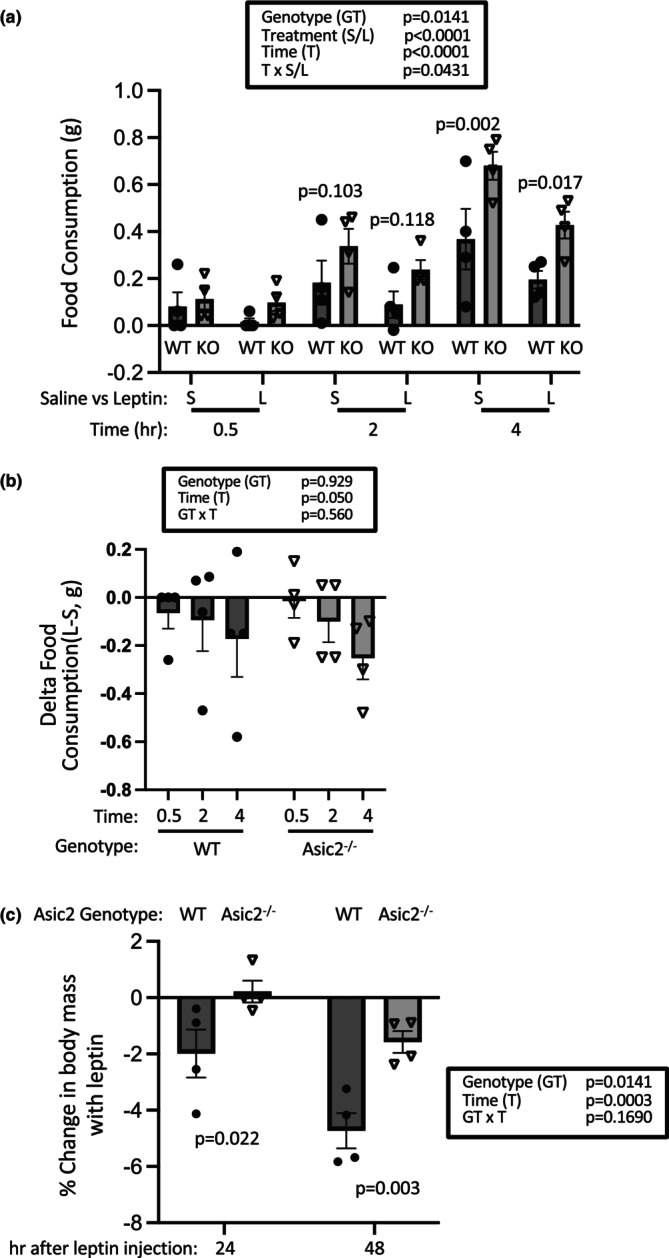
Is leptin sensitivity reduced in Asic2^−/−^ mice? (a) Overall, food consumption was greater in Asic2^−/−^ mice (main effect of genotype *p* = 0.0141) and leptin suppressed food consumption (*p* < 0.0001). (b) The reduction in food consumption between leptin and saline was not different between groups. (c) However, Asic2^−/−^ mice did not lose as much weight as WT mice measured 24 h following the leptin treatments. Sample sizes for the leptin study were *n* = 4 for both genotypes, females only. Food consumption data were analyzed using a three‐ or two‐way repeated measures ANOVA where appropriate. Body weight changes were analyzed using a two‐way repeated measures ANOVA. ANOVA tests were followed by a Fisher's LSD post hoc test; *p* values for main factors, interactions, and apriori group‐to‐group comparisons are shown for all graphs.

A small group of mice (*n* = 4 female, 12–14 weeks of age) was used to examine responses to leptin over a 4 h period (Figure 3a–c). Overall, Asic2^−/−^ mice consumed more food with saline and leptin than WT mice, as supported by the significant effect of genotype (Figure 3a, *p* = 0.014). The 3‐way repeated measures ANOVA suggests that there was an overall effect of leptin versus saline (Figure 3a, *p* < 0.0001). Although we were unable to detect differences in leptin‐suppression of food consumption between genotypes (Figure 3b), likely due to the small sample size and the small food mass consumed during this time frame, Asic2^−/−^ mice lost less weight 24 and 48 h after the first and second leptin injection (Figure 3c). The weight loss 24–48 h post injection reflects a sustained suppressive effect of leptin on food consumption. Thus, the lack of weight loss in the Asic2^−/−^ group may reflect a reduced effect of leptin to inhibit food consumption in the period following leptin injection. These data suggest that Asic2^−/−^ mice may be less sensitive to acute leptin.

### 3.3 Body composition of WT and Asic2^−/−^ before and after 5‐week high‐fat diet

**FIGURE 4:**
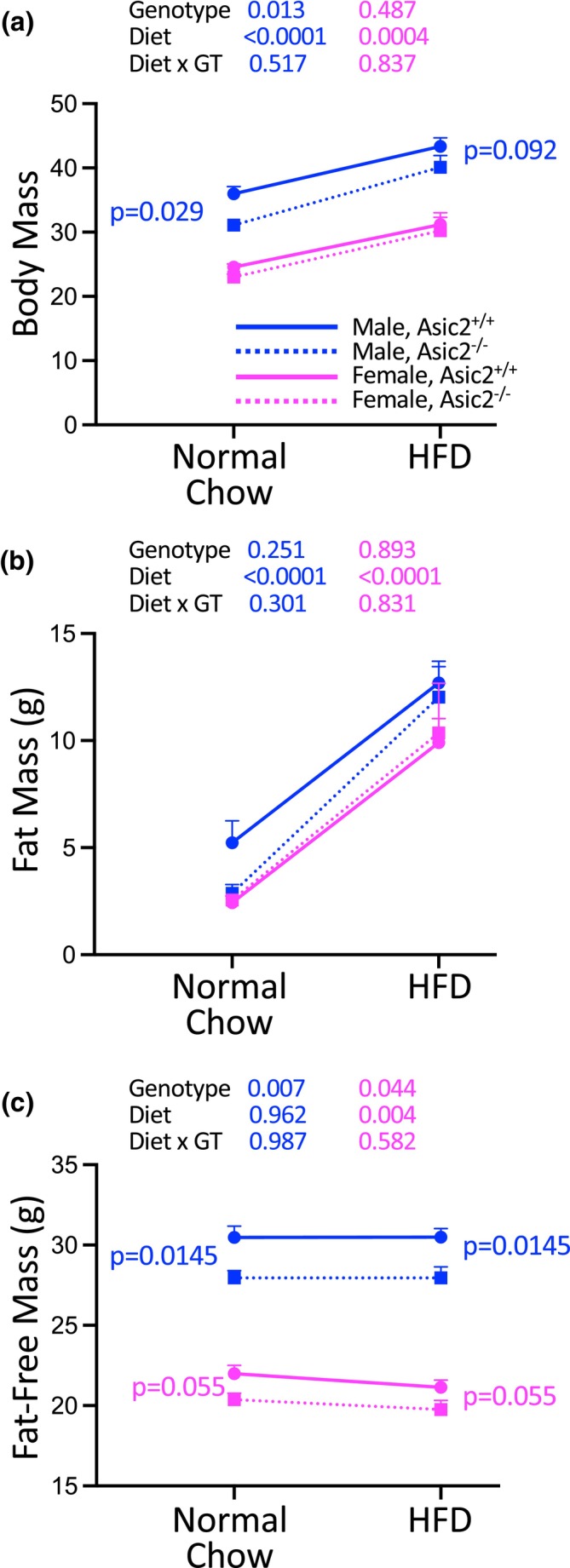
Asic2^−/−^ mice have lower fat‐free mass before and after a 5‐week high‐fat diet. Body composition was measured by Echo MRI at 16 weeks of age and again following 5 weeks of a HFD (60% kcal fat). Body mass (a), fat mass (b), and fat‐free mass (c) are shown in male (blue) and female (magenta), and WT (solid line) and Asic2^−/−^ (dashed line) animals, *n* = 6–7 animals/group. Body mass and fat‐free mass are lower in Asic2^−/−^ male mice, regardless of diet. Fat‐free mass is also lower in Asic2^−/−^ female mice, regardless of diet. Data are shown for male (blue) and female (magenta), and WT (solid line) and Asic2^−/−^ (dashed line) animals, *n* = 6–7 animals/group. Data represent mean ± SEM were analyzed using a repeated two‐way ANOVA for male and female groups, followed by the Fisher Post Hoc test (*p* values for where *p* < 0.200 are shown on panel). HFD, high‐fat diet.

In a separate group of mice, body mass and composition were examined at 16 weeks of age and again following 5 weeks of HFD (Figure 4). Both genotypes gained weight and had similar changes in body composition with HFD. However, Asic2^−/−^ male mice weighed less than WT counterparts regardless of diet. Lower body mass in Asic2^−/−^ mice was not previously reported by our group or others (Bornstein et al., 2024; Fazia et al., 2019; Gannon et al., 2008, 2015; Lin et al., 2024; Lu et al., 2009; Price et al., 2000, 2014). Male and female Asic2^−/−^ mice had lower lean body masses on normal chow and following HFD. Percent fat and fat‐free masses (not shown) were not different between genotypes.

### 3.4 Activity and sleep of WT and Asic2^−/−^ before and after 5‐week high‐fat diet

**FIGURE 5:**
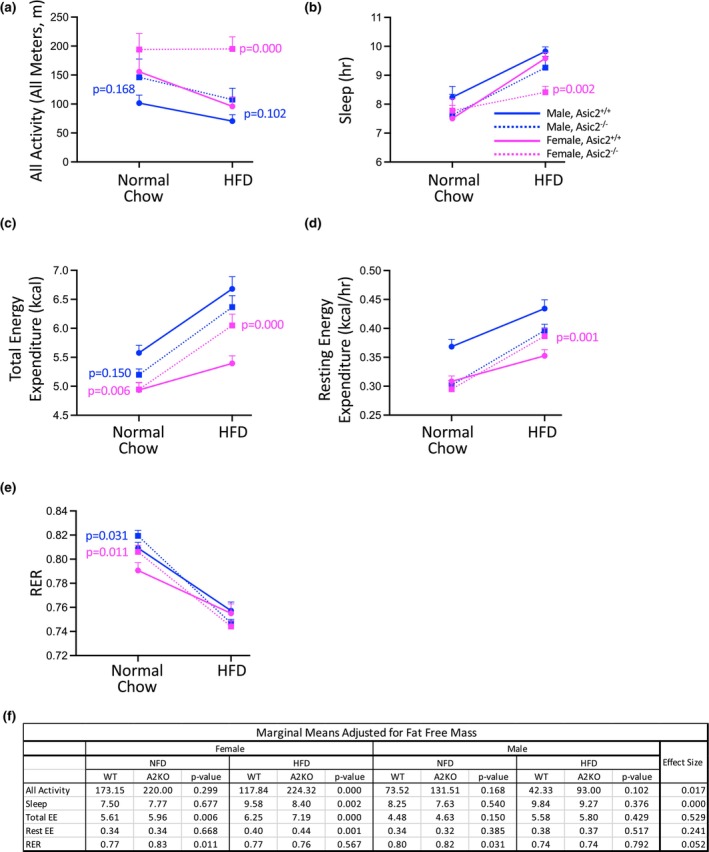
Effect of a normal chow and high fat diet on motor activity, sleep, total and resting energy expenditure, and RER in WT and Asic2^−/−^ mice. Absolute values are shown in (a)–(e) and modified means adjusted for fat‐free mass, *p* values, and effect size are shown in (f). (a) Motor activity was quantified as all movement in X, Y, and Z planes at 16 weeks of age and after a 5‐week HFD. Asic2^−/−^ mice are more active than WT on both diets. Motor activity was greater in male Asic2^−/−^ mice on normal chow. The HFD suppressed motor activity in male and female WT and male Asic2^−/−^ mice, but not in female Asic2^−/−^ mice. (b) Sleep, quantified as lack of movement for >60 s, was not different overall. The HFD increased sleep in both genotypes. Sleep was reduced in female Asic2^−/−^ mice compared to WT following the 5‐week HFD, consistent with the increased activity with HFD in this group. (c) Total energy expenditure was elevated in Asic2^−/−^ females, and trended higher in males, compared to WT counterparts on normal chow. Following HFD total energy expenditure was increased to a greater extent in the Asic2^−/−^ female group. (d) Resting energy expenditure, an estimate of basal metabolic rate, was identical in Asic2^−/−^ female mice while on the normal chow. Resting energy expenditure was increased to in the Asic2^−/−^ female group. (e) The RER was higher in Asic2^−/−^ male and female mice on normal chow. RER was lowered in all groups following the HFD but was not different between genotypes. Data are shown for male (blue) and female (magenta), and WT (solid line) and Asic2^−/−^ (dashed line) animals, *n* = 5–7 animals/group and represent mean ± SEM. (f) Modified means adjusted for fat‐free mass, *p* values, and effect size were calculated using covariate analysis. Fat‐free mass impacted marginal means than body mass. HFD, high‐fat diet; RER, respiratory exchange ratio.

Absolute values are shown in Figure 5 panels (a) and (b), and modified means adjusted for body mass and fat‐free mass are shown in panel (d). Based on the adjusted means in Figure 5f, 24‐h total activity (x, y, and z planes) and sleep were similar in Asic2^−/−^ mice on normal chow (Figure 5a/b/f). Following the HFD, motor activity was higher in females and trended higher in males (*p* = 0.102) Asic2^−/−^ (Figure 5a/f). HFD decreases sleep in female Asic2^−/−^ (Figure 5b/f), consistent with increased motor activity. We detect similar outcomes when data are normalized to fat‐free body mass.

### 3.5 Energy expenditure and respiratory exchange ratio (RER) in WT and Asic2^−/−^ before and after 5‐week high fat diet

Absolute values are shown in Figure 5 panels (c)–(e), and modified means adjusted for body mass and fat‐free mass are shown in panel (f). Based on the adjusted means in Figure 5f, 24 h total and resting energy expenditure values are presented as absolute values in Figure 5c/d and marginal means adjusted for body mass and fat‐free mass (Figure 5f). On the normal chow, total, but not resting, energy expenditure was higher in female and trended higher in male (*p* = 0.150) Asic2^−/−^ mice. When placed on the HFD, total and resting energy expenditure increased only in female Asic2^−/−^ mice. RER was higher in Asic2^−/−^ mice on normal chow, suggesting increased carbohydrate utilization (Figure 5e/f). RER decreased to a similar level with the HFD for all groups reflecting the increased utilization of dietary fat. Except for RER, we detect similar outcomes when data are normalized to fat‐free body mass.

### 3.6 Food intake patterns of WT and Asic2^−/−^ before and after 5‐week high‐fat diet

**FIGURE 6:**
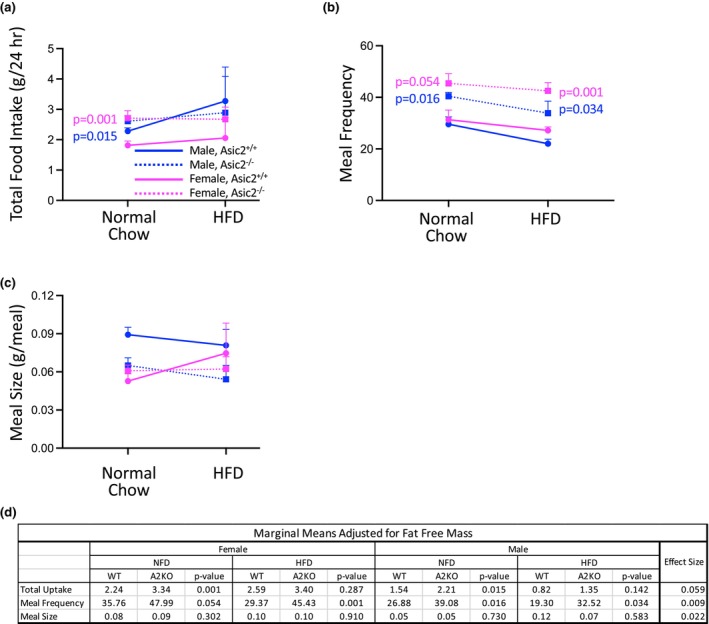
Effect of a normal chow and high fat diet on 24‐h food consumption patterns in WT and Asic2^−/−^ mice. Absolute values are shown in (a)–(c) and modified means adjusted for fat‐free mass, *p* values, and effect size are shown in (d). (a) Asic2^−/−^ mice ate more food in a 24 h period than WT mice when fed normal chow, but not following HFD. (b/c) Meal frequency, but not size, was increased in Asic2^−/−^ mice. Data are shown for male (blue) and female (magenta), and WT (solid line) and Asic2^−/−^ (dashed line) animals, *n* = 5–7 animals/group. Data represent mean ± SEM. (d) Modified means adjusted for fat‐free mass, *p* values, and effect size were calculated using covariate analysis. HFD, high‐fat diet.

Absolute values are shown in Figure 6 panels (a)–(c) and modified means adjusted for body mass and fat‐free mass are shown in panel (d). Based on the adjusted means in Figure 6d, the total 24‐h food intake of Asic2^−/−^ mice was greater on normal chow (Figure 6a/c). Meal frequency, but not size, was increased (Figure 6b–d). Thus, Asic2^−/−^ mice consume more food by eating more frequently.

### 3.7 Circulating insulin, leptin, ghrelin, and glucose are similar in WT and Asic2^−/−^ after a 5‐week high‐fat diet

**FIGURE 7:**
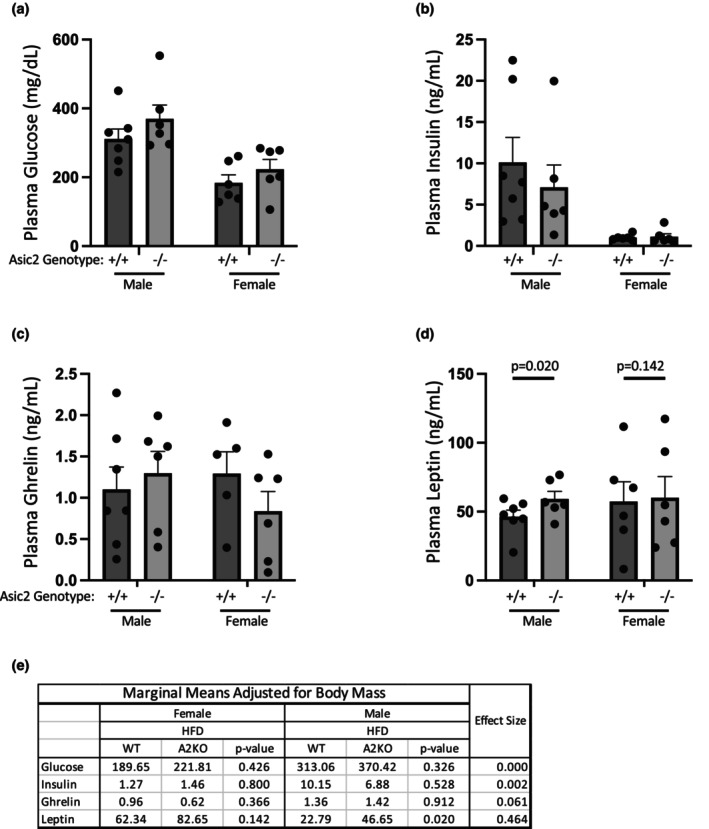
Plasma glucose, insulin, and ghrelin, but not leptin, were not different in WT versus Asic2^−/−^ mice. Plasma samples were collected after 5 weeks on a 60% kcal HFD. Samples were analyzed for glucose (a), insulin (b), ghrelin (c), and leptin (d) using ELISA. Modified means adjusted for fat‐free mass, *p* values, and effect size were calculated using covariate analysis (e). Leptin trended higher in females and was higher in male Asic2^−/−^ mice. Absolute data are represented as mean ± SEM. Samples: plasma + EDTA. Analyzed by Analytic and Assay Core. Plasma leptin was analyzed using Crystal Chem (Cat #90030) ELISA assay. Plasma Glucose was measured using vet axel chemistry analyzer. Plasma insulin was measured using Cat #90080. Plasma ghrelin was measured using Millipore/Sigma Cat #EZRGRT‐91K ELISA.

Absolute values are shown in Figure 7 panels (a)–(d), and modified means adjusted for body mass are shown in panel (e). There were no differences in basal circulating glucose, insulin, or ghrelin. However, leptin was elevated in males (*p* = 0.020) and trended higher in females (*p* = 0.142) Asic2^−/−^ mice following the HFD (Figure 7a–e). Data were obtained from *n* = 6–7 animals per group and analyzed using a 2‐way ANOVA.

### 3.8 Loss of Asic2 does not protect against hepatic steatosis

**FIGURE 8:**
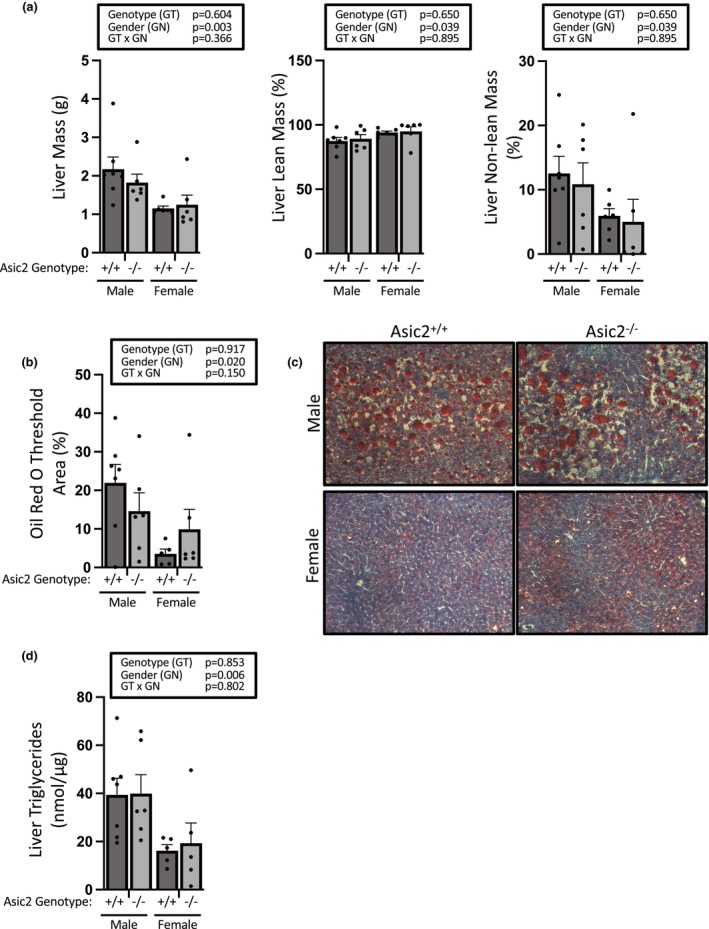
Are Asic2^−/−^ mice protected from hepatic steatosis? (a) Liver mass and Echo MRI quantitation of liver fat content indicates no difference in liver fat content in WT and Asic2^−/−^ mice. (b/c/d) Liver triglyceride content and Oil Red O staining also demonstrate loss of Asic2 does not afford protection. The presence of steatosis is indicated by the elevated liver fat mass, fat droplets visible by microscopy, and liver triglyceride content. Data are mean ± SEM, *n* = 6–7. Data analyzed using two‐way ANOVA, followed by Fishers LSD test, all *p* values shown. No statistical differences between genotypes were found. A covariate analysis was also performed for all data, and no differences were identified.

High‐fat feeding promotes lipid accumulation in the liver. We found no statistical difference in measures of hepatic lipid accumulation in Asic2^−/−^ mice (Figure 8). While liver mass (a, left panel), liver fat masses (a, right panel), Oil Red O staining (b/c), and triglyceride content (d) were lower in females, there was no effect of genotype on either metric (Figure 8a–d). A covariate analysis adjusting means for body mass and fat mass did not change the outcome. Data were obtained from *n* = 6 to 7 animals per group and analyzed using a 2‐way ANOVA.

### 3.9 Morphometric measures were similar between WT and Asic2^−/−^ mice

Kidney, heart, liver, and spleen masses in Asic2^−/−^ mice, adjusted for body and tibial length and body mass following the 5‐week HFD, are shown in Table 2. Covariate analysis revealed significantly lower renal masses (*p* = 0.02) and a trend toward lower heart masses (*p* = 0.111) in males, and a trend toward lower renal masses in females (*p* = 0.138).

**TABLE 2:** Morphometric data from WT and Asic2^−/−^ mice used in the metabolic homeostasis study following the high‐fat diet. Modified means, adjusted for body and fat masses, revealed lower renal mass in males.

Marginal meanss adjusted for body mass, body length, and Tibial length						
	Female			Male		
	HFD			HFD		
	WT	A2KO	*p* Value	WT	A2KO	*p* Value
Heart	0.1358	0.1365	0.906	0.1705	0.1599	0.111
Kidney	0.3261	0.3026	0.138	0.4464	0.4021	0.020
Liver	1.3556	1.4285	0.645	1.7293	1.7475	0.916
Spleen	0.1058	0.1066	0.969	0.1033	0.1032	0.997

## 4 DISCUSSION

### 4.1 Summary

Asic channels are a family of cation channels that are gated by rapid increases in extracellular H^+^ ions. Asic1 and Asic2 expression has been characterized in hippocampus and amygdala neurons (Sherwood et al., 2011; Tong et al., 2008; Zha et al., 2009). While Asic2 expression in the hypothalamus was identified previously (Bassilana et al., 1997; Price et al., 2014), the precise regional expression of Asic2 was unknown. Our analysis of the HypoMap data set has provided insight on Asic2 localization in the hypothalamus. Our findings demonstrate Asic2 is robustly expressed in glutamatergic and gabaergic neurons found in the mamillary, arcuate, lateral, and ventromedial regions. The latter three regions are closely associated with hunger‐satiety, energy expenditure, and glucose homeostasis (Belgardt et al., 2009; Lavoie et al., 2023; Tu et al., 2022). Asic2 is not just expressed in neurons, but also expressed in glial cells (astrocytes, oligodendrocytes, and microglia) which confirms previous reports (Clark et al., 2010; Feldman et al., 2008; Vila‐Carriles et al., 2006, 2007; Xia et al., 2003; Yu et al., 2015). Moreover, global loss of Asic2 has a significant impact on (1) acute weight loss responses to the anorexic hormone leptin, (2) hunger‐satiety‐energy expenditure homeostasis under normal fat and high‐fat feeding, and (3) plasma leptin levels.

### 4.2 Asic2 localization in the arcuate neuron populations

We found Asic2 is expressed in roughly 35% of hypothalamic neurons and 43% of arcuate neurons. Within the arcuate population, co‐expression analysis of the HypoMap data set indicates that Asic2 is expressed in roughly half of the Agrp and Pomc populations (46%–47%). Distilling the colocalization to include Lepr or Insr increases the colocalization of Asic2 in Agrp and Pomc neurons to 60%–73%. These findings suggest Asic2 expression is concentrated in metabolically responsive arcuate neuron populations (Pomc, Agrp, Insr, Lepr). This heterogeneous expression pattern suggests Asic2 is not likely playing a “housekeeping” role but may have a specific function in these neurons.

### 4.3 Phenotype comparison of Asic2^−/−^ mice to Agrp and Pomc knockout mice

Asic2^−/−^ mice share phenotypic characteristics and tendencies with both Agrp (lower body mass, higher energy expenditure, and increased motor activity) and Pomc (increased food consumption) deletion models (Challis et al., 2004; Tong et al., 2008; Wortley et al., 2005) which is not surprising since Asic2 has a similar distribution pattern in these neuron populations (Steuernagel et al., 2022) (Table 3). Since many Agrp and Pomc neurons express the LepR (Lavoie et al., 2023; Steuernagel et al., 2022), we wanted to determine if acute leptin responses were altered in Asic2^−/−^ mice. We found Asic2 mice^−/−^ were protected against acute leptin‐induced weight loss, but not food consumption. The reduced weight loss in the Asic2^−/−^ model is consistent with a role for Asic2 in the anorexic response to leptin. Differences in body weight, but not food consumption, likely reflect the inaccuracy of measuring food consumption as the kibbles may fragment while eating. Understanding the specific contributions of Asic2 in Agrp and Pomc neurons will require the utilization of Pomc‐specific Asic2 knockout mice.

**TABLE 3:** Phenotype comparison of Asic2^−/−^ with Agrp^−/−^, Agrp‐Cre × Vgat^fl/fl^, and Pomc^−/−^ mice. Asic2^−/−^ shares features of reduced body mass, increased activity, and energy expenditure of Agrp‐Cre × Vgat^fl/fl^, and increased food consumption with the Pomc^−/−^ models.

End point (24 h, NFD)	ASIC2^−/−^	Agrp^−/−^ Wortley et al	Agrp‐Cre × Vgat^fl/fl^ Tong et al., Chen et al	Pomc^−/−^ Challis et al
Body mass	↓	↓	↓	↑
Fat mass	↓ (M)	↓		↑ (↑%)
Lean mass	←→	←→		↑ (↓%)
EE or O_2_ uptake/LBM	↑ EE	↑ (O_2_)	↑ (O_2_)	←→
	(O_2_ *p* = 0.099)			
Food intake	↑	←→	↓	↑
Activity	↑	↑	↑	
Food consumption to ghrelin	↑		↑	↑↑
Food consumption to leptin	? (protected from weight loss)			↑

A few other studies have shown motor activity was not different or slightly decreased in male Asic2^−/−^ mice (Bornstein et al., 2024; Jiang et al., 2013). The underlying reasons for the increased activity in our study are unclear but may reflect the testing/housing environment (InfraMOT, VersaMax vs. Sable Promethion). The Sable system continuously measures fine and gross motor activity and includes an elevated, enclosed weight‐sensing “hut” for the mice in standard cage footprint. The InfraMOT measures gross motor activity from an overhead sensor in a standard plexiglass cage, and activity was measured for 48 h (Bornstein et al., 2024). The VersaMax provides an open field environment, and activity was measured for 3 h during the light cycle (Jiang et al., 2013). The data in the current study was collected in two independent trials and have been repeated in a third trial. Our study examined behavior over three 24‐h periods. It is unclear what factor(s) drive the increased motor activity; however, increased food consumption may be a factor.

### 4.4 Is substrate utilization altered in Asic2^−/−^ mice?

At least when fed normal chow, RER was greater in Asic2^−/−^ mice, indicating a greater utilization of carbohydrates compared to WT. Although motor activity was not statistically greater on normal chow, the adjusted motor activity was increased ~50% in the Asic2^−/−^ (the small sample size and variability of motor activity may have contributed to this) which may contribute to greater carbohydrate utilization in Asic2^−/−^ groups. The HFD reduced the RER to similar values in both genotypes, indicating that fat utilization was enhanced as expected.

### 4.5 What is the role of Asic2 in arcuate neurons?

Our current understanding of Asic2 suggests it serves a modulatory role in synaptic plasticity, maintaining synapses and dendritic spines (Li et al., 2012). The HypoMap expression pattern of Asic2 lends itself to several interpretations. First, that Asic2 message is expressed in neurons in most hypothalamic regions suggests its role is probably not specific to regulating hunger‐satiety. Second, that Asic2 is expressed in 43% of the arcuate neuron population implies it is probably not playing a “housekeeping” role; rather, it is playing a specific role. However, that specific role is unclear. One possible mechanism of action is that pre‐ or post‐synaptic Asic channels may be gated by H^+^ ions released when the synaptic vesicle fuses with the presynaptic membrane to regulate the excitability of neighboring receptors (Du et al., 2014; Hill & Ben‐Shahar, 2018; Zhu et al., 2021). The pH of synaptic vesicles of ~5.5 (Ahdut‐Hacohen et al., 2004; Sinning & Hubner, 2013) is close to the pEC_50_ for Asic2 homomeric channels (4.1–5.0) and Asic1/2 hybrid channels (4.8–5.7) (Bassilana et al., 1997; Joeres et al., 2016). Thus, Asic2 may play a role in the potentiation of synaptic transmission.

### 4.6 Significance and limitations

This study provides compelling evidence that Asic2 contributes to metabolic homeostasis, including feeding‐satiety and motor activity. However, there are limitations associated with our study. First, we cannot conclude with confidence that the arcuate region neurons mediate the metabolic and behavioral phenotype of Asic2^−/−^ mice. It is unlikely that loss of arcuate Asic2 is exclusively responsible for the Asic2^−/−^ phenotype. Other hypothalamic regions expressing Asic2, such as the ventromedial hypothalamus and paraventricular region, also contribute to metabolic homeostasis. The influence of non‐neuronal central Asic2 cannot be excluded either. For example, astrocytes play an important role in glucose regulation in the ventromedial hypothalamus (Tu et al., 2022). Second, animals were not housed at thermoneutrality (28–32°C) which can be a confounding factor in the development of obesity (Fischer et al., 2018). Third, our normal chow was not the ideal control for the HFD. Thus, the switch from normal chow to HFD may introduce more variables than the macronutrients. Fourth, although WT and knockout lines were generated from Asic2 heterozygous mating pairs, they were maintained as homozygous mating pairs, raising the possibility that a genetic variant may contribute to the phenotype. Future studies using conditional Asic2 deletion in specific neuron populations (± thermoneutrality housing environment) will be required to address the physiological importance of regional hypothalamic Asic2. In closing, our findings establish a novel role for Asic2 and other degenerin channels in metabolic homeostasis and the potential for targeting central Asic channels in obesity and obesity‐related diseases.

## AUTHOR CONTRIBUTIONS

JS, JK, LC, and HAD assisted with Seurat analysis of the HypoMap data set, drafted methods, and edited the manuscript. MH, JMC, EH, and EB assisted with data collection and edited the manuscript. HAD and MH conceived of experiments, data collection, and data analysis, prepared figures, drafted, and edited the manuscript. SL conducted covariate analyses.

## FUNDING INFORMATION

Research reported in this publication was supported by the NIH P30GM149404 and P20GM104357, which help support the Imaging Core, Metabolic Phenotyping Core, and Analytic and Assay Core Facilities at the University of Mississippi Medical Center used in the completion of this work. P20GM144041 provided support for JK and LC. Other support provided by 5T32HL105324 (MH), P20GM121334 (HAD, JMC), R01HL163076 (JMC), R01DK124327 (JS), P20FM121334, and R01DK137167 (HAD) also provided personnel support.

## ETHICS STATEMENT

All animal protocols were approved by the University of Mississippi Medical Center‘s IACUC. Photographic images were unaltered from orignal collection.
